# Effects of prehospital management in out-of-hospital cardiac arrest: advanced airway and adrenaline administration

**DOI:** 10.1186/s12913-022-07890-x

**Published:** 2022-04-23

**Authors:** Yu Wang, Qun Zhang, Guang Bo Qu, Fang Fang, Xiao Kang Dai, Liang Xi Yu, Hong Zhang

**Affiliations:** 1 Department of Emergency Medicine, The First Affiliated Hospital of Anhui Medical University, Hefei City, Anhui Province China; 2 Hefei Emergency Center, Hefei City, Anhui Province China; 3 Anhui Medical University, Hefei City, Anhui Province China

**Keywords:** Advanced airway management, Adrenaline, Out-of-hospital cardiac arrest, Prehospital

## Abstract

**Background:**

There is uncertainty about the best approaches for advanced airway management (AAM) and the effectiveness of adrenaline treatments in Out-of-hospital cardiac arrest (OHCA). This study aimed to evaluate whether AAM and adrenaline administration provided by Emergency Medical Service (EMS) can improve the outcomes of OHCA.

**Methods:**

This study was a prospective analysis of collected data based on OHCA adult patients treated by the EMS in China from January 2019 to December 2020.The patients were divided into AAM group and no AAM group, and into subgroups according to whether adrenaline was used. The outcome was rate of return of spontaneous circulation (ROSC), survival to admission and hospital discharge.

**Results:**

1533 OHCA patients were reported. The probability of ROSC outcome and survival admission in the AAM group was significantly higher, compared with no AAM group. The probability of ROSC outcome in the AAM group increased by 66% (adjusted OR: 1.66, 95%CI, 1.02–2.71). There were no significant differences in outcomes between the adrenaline and no adrenaline groups. The combined treatment of AAM and adrenaline increased the probability of ROSC outcome by 114% (adjusted OR, 2.14, 95%CI, 1.20–3.81) and the probability of survival to admission increased by 115% (adjusted OR, 2.15, 95%CI, 1.16–3.97).

**Conclusions:**

The prehospital AAM and the combined treatment of AAM and adrenaline in OHCA patients are both associated with an increased rate of ROSC. The combined treatment of AAM and adrenaline can improve rate of survival to admission in OHCA patients.

## Introduction

OHCA with high incidence and low survival rate is one of the most important public health issues. More than 230 million people suffer from cardiovascular diseases and about 550,000 people suffer from cardiac arrest every year in China [1]. Cardiac arrest survival rates vary widely from 0.6 to 25% in the world [2, 3].

In recent years, there has been increasing recognition of the prehospital management of cardiac arrest. Successful resuscitation relies on a strong chain of survival with the community, dispatch centre, ambulance, and hospital working together [4]. EMS aims to improve response times, respond to dispatch calls as timely as possible and assure cardiopulmonary resuscitation (CPR) quality. EMS personnel often provide advanced life support to patients with OHCA on scene. Therefore, continuing to improve prehospital management to increase the survival rate of OHCA patients is still a topic of emergency and critical scholars around the world.

However, there is uncertainty about the best approaches for prehospital airway management in OHCA. Conflicting results were produced when comparing advanced (eg, intubation or supraglottic airways) with basic airways (eg, bag–valve–mask) [5–7]. The inconsistent effects of prehospital AAM in OHCA were influenced by multifaceted, unmeasured factors such as the timing of AAM or the proficiency of EMS personnel. A study reported regional variation in the effects of prehospital AAM on outcomes of OHCA patients from Osaka (Japan), Seoul (Republic of Korea), Singapore (Singapore), and Taipei (Taiwan) between 2012 and 2014 [8].

The adrenaline to be administered during cardiac arrest is recommended in 2010 American Heart Association Guidelines for Cardiopulmonary Resuscitation and Emergency Cardiovascular Care [9]. However there is controversy about adrenaline administration in OHCA. A retrospective study found adrenaline administration is associated with an increase of ROSC and with improvement in the neurological outcome on which EMSs’ CPR duration is performed between 15 and 19 min [10]. No evidence that drugs improved outcomes was found in the results of two landmark studies [11, 12].

This study reported the 2019–2020 data of OHCA patients in Hefei, China as a base line, summarized patient characteristics, processes and outcomes for OHCA. Information was also provided on the optimal approach to AAM and adrenaline administration in OHCA. This study aimed to evaluate whether the prehospital AAM and adrenaline administration provided by EMS improved the outcomes. It is hoped to explore the epidemiological characteristics of OHCA in Hefei, the problems in regional EMS response and interventions, so as to improve the quality of prehospital CPR and the survival rate of OHCA patients.

## Methods

### Study design

This study was a prospective analysis of collected data based on OHCA patients treated by the Emergency Center in Hefei China from January 2019 to December 2020. The registry database included the OHCA patients’ demographic characteristics, as well as EMS and Emergency Department (ED) information based on the standardized Utstein style.

### EMS characteristics and procedures

The Hefei Emergency Center serves a population of 5,118,200 [13]. It includes the Dispatching Department of Hefei Emergency Center and 20 sub-centers and 42 emergency stations established relying on medical institutions. All emergency personnel have the basic CPR training and passed the examination of Hefei Emergency Center and have legal practice qualification. Three or four EMS workers constitute the ambulance team, including a doctor, a driver and a nurse or an emergency personnel assistant.

The Dispatching Department of Hefei Emergency Center obtains the patient information through the emergency service number (120). After arriving at the scene, the doctor decides whether to make the CPR according to the patient’s situation, CPR is provided by the EMS personnel on scene, and then the patient is transferred to the hospital for stabilization and definitive management .

When cardiac arrest is diagnosed, chest compression and ventilation using a bag-valve mask are immediately initiated, and CPR is provided by the EMS personnel according to 2015 American Heart Association CPR and ECC guidelines [14]. If necessary, the doctor applies a semiautomated external defibrillator and AAM. After attempting defibrillation, inserting an advanced airway device, the patient is provided with advanced life support, including the administration of adrenaline by the doctor before arrival at the hospital.

### Sample size

The needed sample size was calculated using the formula of sample size calculation of the experimental epidemiological study: N = [*Z*_α_ $\sqrt{2P\left(1-P\right)}$ +*Z*_β_ $\sqrt{P_c\left(1-{P}_c\right)+{P}_e\left(1-{P}_e\right)}$]^2^/(*P*_*c*_- *P*_*e*_)^2^_._ In this formula, *P*_*c*_ represented for the probability of outcome among control group, *P*_*e*_ represented for the probability of outcome among experimental group, *P* = (*P*_*c*_ + *P*_*e*_)/2. We referred the probability of ROSC among AAM and no AAM groups [15], and set α = 0.05, β = 0.10. Finally, the required minimum sample size was 92.

### Patient selection

#### Inclusion criteria

The patients in this study were selected from among all adult patients who had experienced OHCA and were subsequently treated with CPR and transported to a medical institution by EMS personnel in Hefei, China between 1 January 2019 and 31 December 2020.

#### Exclusion criteria

Patients under 18 years of age; patients with spontaneous circulation had been restored before the arrival of EMS personnel and patients who had a ‘Do Not Attempt Resuscitation’(DNAR) decision; patients whose medical records were missing data and for whom more than 60 min from the emergency call to the initiation of CPR were excluded.

### Ethical approval

The Ethics Committee of Qilu Hospital of Shandong University granted ethics approval, reference number 2019012.

### Exposures

The primary exposure of the study was prehospital AAM. AAM was defined as an invasive technique used for airway management, including intu­bation and all types of supraglottic airways. The no AAM group included patients who underwent a non-invasive technique for airway management, such as use of a bag valve mask, with or without the inclusion of the nasopharyngeal and/or oropharyngeal airways.

The second exposure of the study was administration of adrenaline. After basic life support the EMS personnel attempt to gain peripheral venous access to administer 1 mg of adrenaline intravenously every 3 to 5 min until the ROSC or arrival at the hospital.

### Outcomes

The primary outcome was the probability of prehospital ROSC and ROSC at ED among all included patients. The second outcome was the probability of survival to admission and survival to hospital discharge among all included patients.

### Data collection

The study is a part of the project of the incidence rate, mortality and risk factors of cardiac arrest in Chinese population which is a special project of science and technology basic resources investigation by Qilu Hospital of Shandong University.

Prehospital emergency medical records were collected through the database of Hefei Emergency Center as the Sub center of the project. Patient prognosis follow-up data is collected through the hospital where the patient were admission.

The registry included data on the OHCA patients’ socio-demographic characteristics, as well as EMS and ED information based on the standardized Utstein style, such as circumstances of the OHCA (witness status, bystander CPR), electrocardiogram (ECG) rhythm at cardiac arrest, EMS time intervals, On-site treatment measures (tracheal intubation, electrical defibrillation, adrenaline use, etc.)and patient outcomes.

### Statistical analysis

The continuance data was present as mean and standard deviation (Mean ± SD) and the counting data was described as number and percentage (n,%). Missing values in EMS Scene rescue time and emergency transport rescue time were filled with multiple imputation [16]. The comparison of EMS response time and age between different groups using the independent *t* test. The comparison of the rate of ROSC, Survival to admission, and survival to hospital discharge between different groups using chi-square test. To explore the effects of advanced airway and adrenaline and their combined effect on outcomes of ROSC, survival to hospital admission and survival to hospital discharge, logistic regression models were constructed with or without adjusting confounders which including age, sex, prehospital time, electrical defibrillation, origin of cardiac arrest, bystander CPR and witness. All statistical analyses were performed using SPSS software 23.0, and *P* value < 0.05 was considered to be statistically significant.

## Result

### Characteristics of participants

**Fig. 1:**
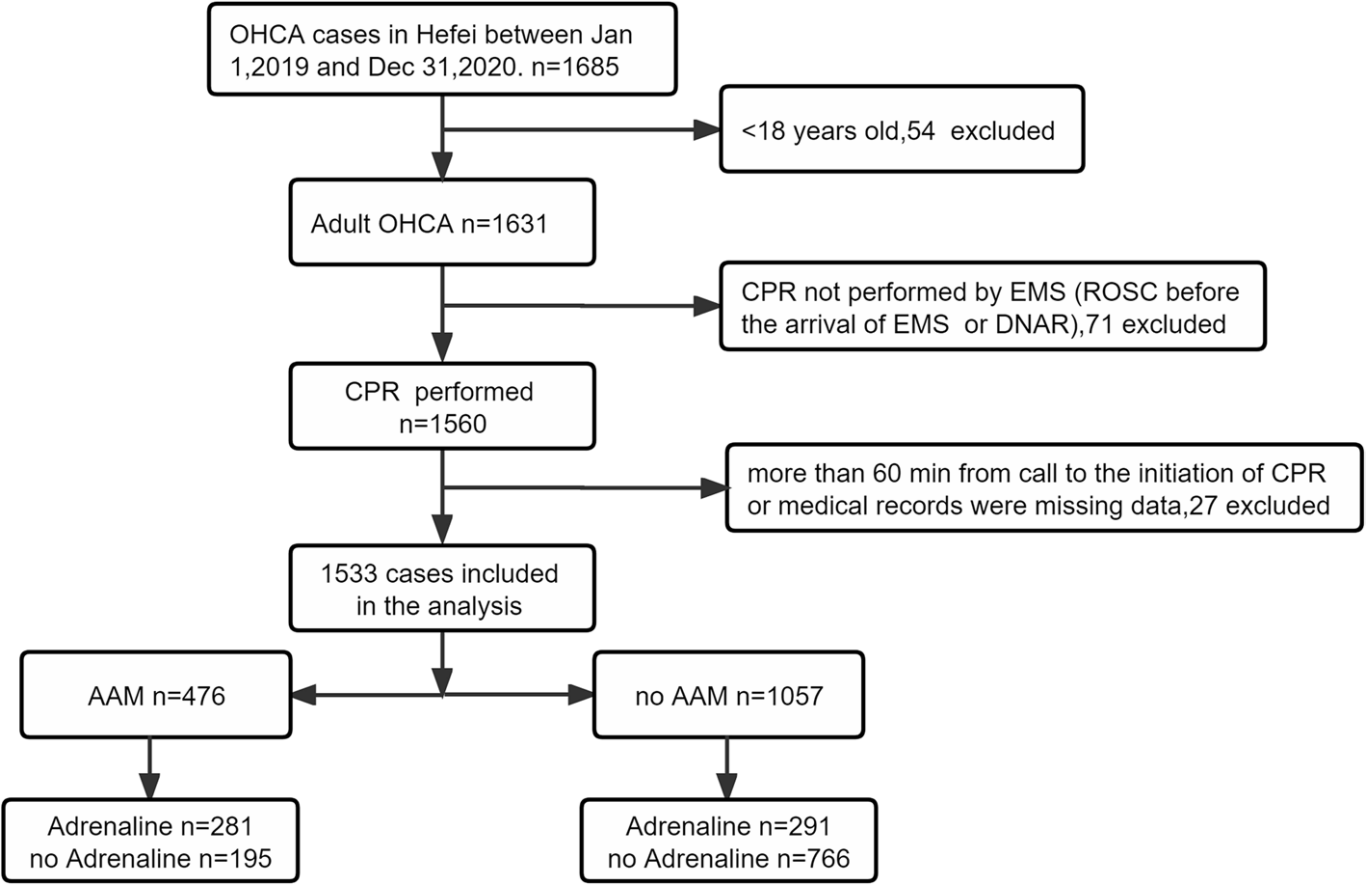
Study participant selection

In this study, 1685 people occurred with OHCA from January 2019 to December 2020, and after excluding patients who met exclusion criteria of patient selection, 1533 patients were finally included (Fig. 1), with an average age of 64.26 ± 17.65 years old, including 1030 males, accounting for 67.2% For the causes of CPR, there were 1006 patients with cardiac origin, accounting for 65.6%, 90 patients had ventricular fibrillation, accounting for 5.9%. Among all included patients, 476 (31.1%) were treated with advanced airway and 572 (37.3%) were treated with adrenaline. For patients outcomes, 90 (5.9%) patients had ROSC, 77 (5.0%) patients were survived to hospital admission, and 27 (1.8%) patients survived to hospital discharge .

There was no significant difference in initial ECG and bystander CPR between AAM group and no AAM group (Table 1). The characteristics of participants were displayed in Table 1.

**Table 1:** Characteristics of all patients and comparison between AAM and no AAM groups

Variables	All patients (*n* = 1533)	AAM (*n* = 476)	No AAM (*n* = 1057)	*P*
Age (years, Mean ± SD)	64.26 ± 17.65	64.23 ± 17.00	64.28 ± 17.95	0.958
Gender (n, %)				
Male	1030 (67.2)	318 (66.8)	712 (67.4)	0.831
Female	503 (32.8)	158 (33.2)	345 (32.6)	
Origin of cardiac arrest				
Cardiac origin	1006 (65.6)	334 (68.9)	672 (64.1)	< 0.001
Non cardiac	324 (21.1)	104 (21.8)	220 (20.8)	
Trauma	110 (7.2)	15 (3.2)	95 (9.0)	
Other exogenous origin	93 (6.1)	23 (4.8)	70 (6.6)	
Initial ECG (n, %)				
F/VT	90 (5.9)	31 (6.5)	59 (5.6)	0.751
Asystole	1329 (86.7)	411 (86.3)	918 (86.8)	
PEA	114 (7.3)	34 (7.2)	80 (7.6)	
Bystander CPR (n, %)				
Yes	9 (0.6)	3 (0.6)	6 (0.6)	1.000
No	1524 (99.4)	473 (99.4)	1051 (99.4)	
Season of event (n, %)				
Spring	329 (21.5)	97 (20.4)	232 (21.9)	0.108
Summer	364 (23.7)	110 (23.1)	254 (24.0)	
Autumn	405 (26.4)	145 (30.5)	260 (24.6)	
Winter	435 (28.4)	124 (26.1)	311 (29.4)	
EMS response time (minute, Mean ± SD)				
Response time to call for help	1.72 ± 1.54	1.68 ± 1.41	1.74 ± 1.59	0.429
Emergency response time	11.11 ± 5.51	10.77 ± 5.46	11.26 ± 5.53	0.109
EMS Scene rescue time	14.74 ± 11.60	19.09 ± 13.48	12.77 ± 10.06	< 0.001
Emergency transport rescue time	10.05 ± 8.76	10.84 ± 10.56	9.68 ± 7.80	0.032
Total rescue time	24.78 ± 14.50	29.93 ± 16.74	22.46 ± 12.71	< 0.001
Prehospital time	35.89 ± 15.77	40.71 ± 18.09	33.72 ± 14.08	< 0.001
Electrical defibrillation (n, %)				
Yes	36 (2.3)	16 (3.4)	20 (1.9)	0.079
No	1497 (97.7)	460 (96.6)	1037 (98.1)	
Adrenaline (n, %)				
Yes	572 (37.3)	281 (59.0)	291 (27.5)	< 0.001
No	961 (62.7)	195 (41.0)	766 (72.5)	
Witness (n, %)				
Yes	707 (46.1)	205 (43.1)	502 (47.5)	0.108
No	826 (53.9)	271 (56.9)	555 (52.5)	
Outcomes (n, %)				
ROSC	90 (5.9)	38 (8.0)	52 (4.9)	0.018
Survival to admission	77 (5.0)	32 (6.7)	45 (4.3)	0.041
Survival to hospital discharge	27 (1.8)	9 (1.9)	18 (1.7)	0.796

*VF* Ventricular fibrillation, *VT* Ventricular tachycardia, *EMS* Emergency system, *ROSC* Return of spontaneous circulation, *AAM* Advanced airway management, *PEA* Pulseless electrical activity

### The comparison of survival outcomes

**Fig. 2:**
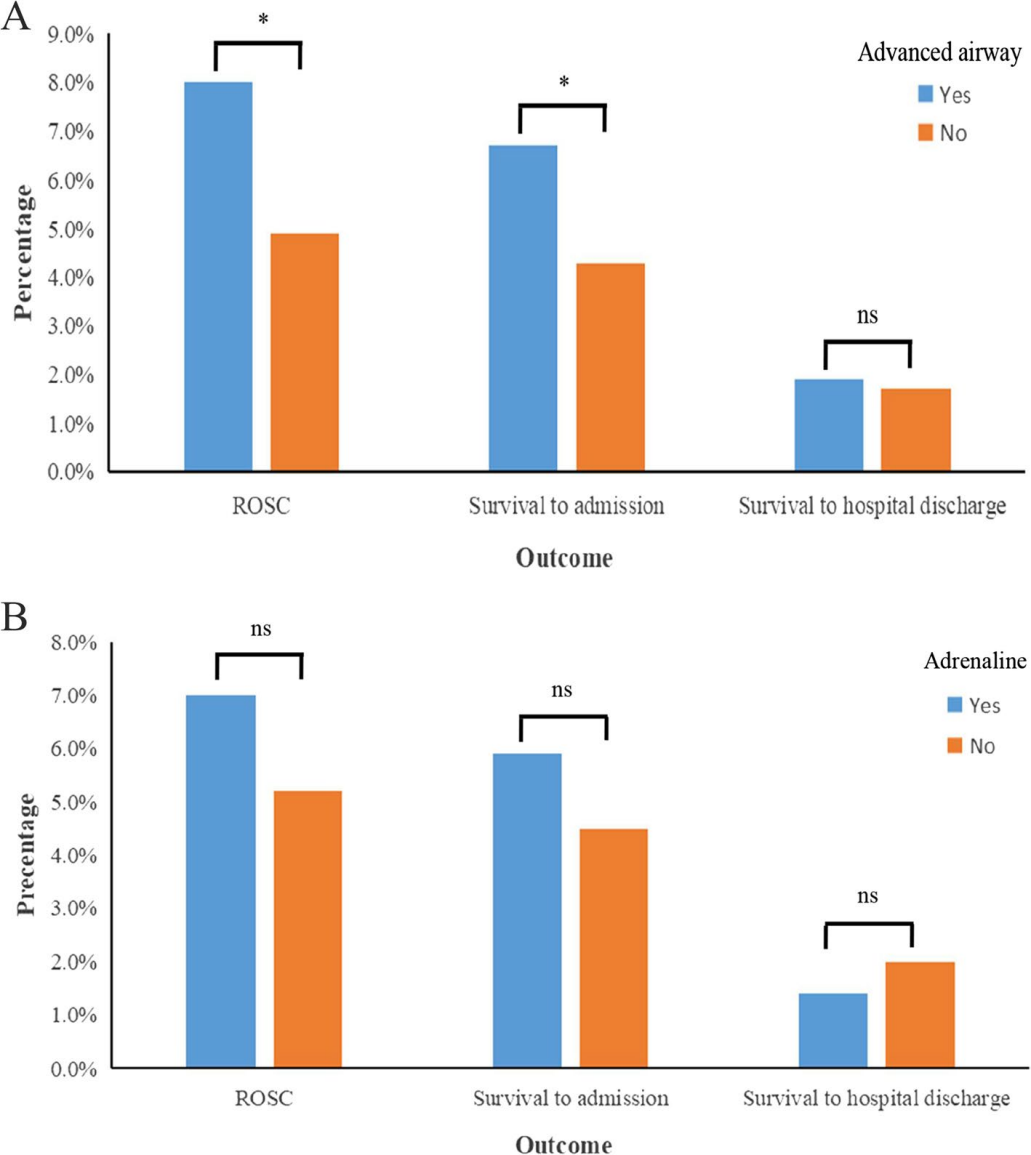
The comparison of survival outcomes in patients with AAM and no AAM

Compared with no AAM group, AAM group has significant higher probability of ROSC and survival to admission, but no difference in the probability of survival to hospital discharge (Fig. 2A and Table 2). However, no significant difference in the probability of ROSC, survival to admission, and survival to hospital discharge between adrenaline group and no adrenaline group (Fig. 2B and Table 2). In addition, there were significant differences in the probability of ROSC, survival to admission, and survival to hospital discharge between rescue scene with and without witness (Table 2).

**Table 2:** Comparison of population characteristics between different outcomes

Variables	ROSC		*P*	Survival to admission		*P*	Survival to hospital discharge P		*P*
	Yes (*n* = 90)	No (*n* = 1443)		Yes (*n* = 77)	No (*n* = 1456)		Yes (*n* = 27)	No (*n* = 1506)	
Age (years, Mean ± SD)	61.66 ± 17.06	64.42 ± 17.68	0.149	63.06 ± 15.46	64.33 ± 17.77	0.542	59.33 ± 16.51	64.35 ± 17.67	0.143
Gender (n, %)									
Male	55 (61.1)	975 (67.6)	0.206	46 (59.7)	984 (67.6)	0.153	17 (63.0)	1013 (67.3)	0.637
Female	35 (38.9)	468 (32.4)		31 (40.3)	472 (32.4)		10 (37.0)	493 (32.7)	
Origin of cardiac arrest									
Cardiac origin	54 (60.0)	952 (66.0)	0.304	47 (61.0)	959 (65.9)	0.291	17 (63.0)	989 (65.7)	0.877
Trauma	6 (6.7)	104 (7.2)		4 (5.2)	106 (7.3)		2 (7.4)	108 (7.2)	
Other exogenous origin	4 (4.4)	89 (6.2)		3 (3.9)	90 (6.2)		1 (3.7)	92 (6.1)	
Non cardiac	26 (28.9)	298 (20.7)		23 (29.9)	301 (20.7)		7 (25.9)	317 (21.0)	
Initial ECG (n, %)									
VF/VT	7 (7.8)	83 (5.8)	< 0.001	5 (6.5)	85 (5.8)	< 0.001	1 (3.7)	89 (5.9)	0.023
Asystole	65 (72.2)	1264 (87.6)		56 (72.7)	1273 (87.4)		20 (74.1)	1309 (86.9)	
PEA	18 (20.0)	96 (6.7)		16 (20.8)	98 (6.7)		6 (22.2)	108 (7.2)	
Bystander CPR (n, %)									
Yes	0 (0.0)	9 (0.6)	1.000	0 (0.0)	9 (0.6)	1.000	0 (0.0)	9 (0.6)	1.000
No	90 (100.0)	1434 (99.4)		77 (100.0)	1447 (99.4)		27 (100.0)	1497 (99.4)	
Season of event (n, %)									
Spring	20 (22.2)	309 (21.4)	0.026	16 (20.8)	313 (21.5)	0.155	10 (37.0)	319 (21.2)	0.235
Summer	31 (34.4)	333 (23.1)		24 (31.2)	340 (23.4)		6 (22.2)	358 (23.8)	
Autumn	24 (26.7)	381 (26.4)		23 (29.9)	382 (26.2)		6 (22.2)	399 (26.5)	
Winter	15 (16.7)	420 (29.1)		14 (18.2)	421 (28.9)		5 (18.5)	430 (28.6)	
EMS response time (minute, Mean ± SD)									
Response time to call for help	1.63 ± 0.88	1.73 ± 1.57	0.570	1.62 ± 0.90	1.73 ± 1.56	0.561	1.56 ± 0.58	1.73 ± 1.55	0.569
Emergency response time	10.33 ± 4.80	11.16 ± 5.55	0.169	9.99 ± 4.63	11.17 ± 5.55	0.067	8.85 ± 2.90	11.15 ± 5.54	< 0.001
EMS Scene rescue time	17.07 ± 13.89	14.59 ± 11.43	0.101	16.27 ± 13.66	14.65 ± 11.48	0.310	16.56 ± 13.61	14.70 ± 11.57	0.488
Emergency transport rescue time	8.33 ± 6.08	10.15 ± 8.89	0.056	8.29 ± 6.01	10.14 ± 8.87	0.012	7.00 ± 5.50	10.10 ± 8.80	0.068
Total rescue time	25.40 ± 15.98	24.74 ± 14.41	0.704	24.56 ± 15.70	24.79 ± 14.44	0.891	23.56 ± 14.96	24.80 ± 14.50	0.658
Prehospital time	35.73 ± 17.28	35.90 ± 15.68	0.929	34.55 ± 16.95	35.96 ± 15.71	0.443	32.41 ± 15.37	35.95 ± 15.77	0.247
Electrical defibrillation (n, %)									
Yes	7 (7.8)	29 (2.0)	0.002	4 (5.2)	32 (2.2)	0.191	2 (7.4)	34 (2.3)	0.131
No	83 (92.2)	1414 (98.0)		73 (94.8)	1424 (97.8)		25 (92.6)	1472 (97.7)	
AAM									
Yes	38 (42.2)	438 (30.4)	0.018	32 (41.6)	444 (30.5)	0.041	9 (33.3)	467 (31.0)	0.796
No	52 (57.8)	1005 (69.6)		45 (58.4)	1012 (69.5)		18 (66.7)	1039 (69.0)	
Adrenaline (n, %)									
Yes	40 (44.4)	532 (36.9)	0.149	34 (44.2)	538 (37.0)	0.203	8 (29.6)	564 (37.5)	0.405
No	50 (55.6)	911 (63.1)		43 (55.8)	918 (63.0)		19 (70.4)	942 (62.5)	
Witness (n, %)									
Yes	62 (68.9)	645 (44.7)	< 0.001	52 (67.5)	655 (45.0)	< 0.001	18 (66.7)	689 (45.8)	0.031
No	28 (31.1)	798 (55.3)		25 (32.5)	801 (55.0)		9 (33.3)	817 (54.2)	

*VF* Ventricular fibrillation, *VT* Ventricular tachycardia, *EMS* Emergency system, *ROSC* Return of spontaneous circulation, *AAM* Advanced airway management, *PEA* Pulseless electrical activity

### Association between advanced airway and adrenaline and risk of survival outcomes

Overall, in AAM group, after adjusting the gender and age of patients, the total time before admission, defibrillation, adrenaline, origin of cardiac arrest, initial ECG, bystander CPR, and witnesses, the probability of ROSC increased by 66% (adjusted OR: 1.66, 95% CI: 1.02–2.71), survival to admission increased by 62% (adjusted OR: 1.62, 95% CI: 0.96–2.73) with not statistical significant, the probability of survival to discharge increased, but it was not significant (adjusted OR,1.26, 95% CI,0.52–3.07) (Table 3).

**Table 3:** The effect of AAM on risk of resuscitation among all patients and adrenaline subgroups

	Crude OR (95%CI)	*P*	Adjusted OR (95%CI)	*P*
**All Patients**^a^				
ROSC	1.67 (1.09–2.59)	0.019	1.66 (1.02–2.71)	0.040
Survival to admission	1.62 (1.02–2.58)	0.043	1.62 (0.96–2.73)	0.068
Survival to hospital discharge	1.11 (0.50–2.49)	0.796	1.26 (0.52–3.07)	0.614
**Adrenaline**^b^				
ROSC	2.02 (1.03–3.95)	0.041	1.71 (0.82–3.55)	0.150
Survival to admission	1.97 (0.96–4.07)	0.065	1.81 (0.83–3.92)	0.134
Survival to hospital discharge	NA	NA	NA	0.994
**No adrenaline**^b^				
ROSC	1.26 (0.64–2.45)	0.504	1.57 (0.77–3.21)	0.212
Survival to admission	1.20 (0.58–2.48)	0.621	1.48 (0.69–3.19)	0.317
Survival to hospital discharge	0.21 (0.03–1.61)	0.135	0.22 (0.03–1.96)	0.177

^a^Adjust gender, age, total rescue time, defibrillation, adrenaline, origin of cardiac arrest, initial ECG, bystander CPR, witness

^b^Adjust gender, age,total rescue time, defibrillation, origin of cardiac arrest, initial ECG, bystander CPR, witness

In addition, subgroup analysis was performed according to whether the patients were further given adrenaline, AAM increased the probability of ROSC (adjusted OR: 1.71, 95%CI: 0.82–3.55), survival to admission increased (adjusted OR: 1.81, 95% CI: 0.83–3.92), but not statistically significant (Table 3).

Results of interaction analysis showed that only AAM or adrenaline treatment had no significant effect on the occurrence of patient outcomes, but the combination of AAM and adrenaline treatment significantly increased the probability of ROSC and survival to admission, no matter whether the confounding factors were adjusted or not (Table 4).

**Table 4:** The joint effect of advanced airway and adrenaline on risk of ROCS, survival to admission, and survival to hospital discharge

Outcome	Group	Crude OR (95%CI)	*P*	Adjusted OR (95%CI)	*P*
**ROSC**	Neither adrenaline nor AAM	reference		reference	
	AAM and no adrenaline	1.26 (0.64–2.45)	0.504	1.36 (0.68–2.72)	0.392
	Adrenaline and no AAM	0.97 (0.52–1.81)	0.920	1.04 (0.54–1.99)	0.917
	Both adrenaline and AAM	1.95 (1.16–3.28)	0.011	2.14 (1.20–3.81)	0.010
**Survival to admission**	Neither adrenaline nor AAM	reference		reference	
	AAM and no adrenaline	1.21 (0.58–2.48)	0.621	1.31 (0.62–2.77)	0.476
	Adrenaline and no AAM	0.95 (0.49–1.88)	0.894	1.06 (0.53–2.13)	0.865
	Both adrenaline and AAM	1.89 (1.08–3.30)	0.026	2.15 (1.16–3.97)	0.015
**Survival to hospital discharge**	Neither adrenaline nor AAM	reference		reference	
	AAM and no adrenaline	0.21 (0.03–1.61)	0.135	0.19 (0.02–1.58)	0.124
	Adrenaline and no AAM	NA	0.994	NA	0.994
	Both adrenaline and AAM	1.22 (0.52–2.83)	0.647	1.33 (0.52–3.40)	0.552

Adjust gender, age,total rescue time, defibrillation, origin of cardiac arrest, initial ECG, bystander CPR, witness

After adjusting for gender, age, total time, defibrillation, cardiogenic and eyewitness conditions, the AAM and adrenaline group increased the probability of ROSC by 114% (adjusted OR: 2.14, 95%CI, 1.20–3.81), survival to admission increased by 115% (adjusted OR: 2.15, 95%CI, 1.16–3.97). No significant effect of combined treatment of AAM and adrenaline on probability of survival to hospital discharge was found (Table 4).

## Discussion

Improving links in the cardiac arrest chain of survival can improve outcomes, particularly the early links in this chain. EMS has a central role in the coordination of the response to OHCA,and its prehospital managements have the greatest impact on survival. Until recently, regional variations in prehospital AAM did not attract the attention of researchers in China. This study showed **t**he prehospital AAM and the combined treatment of AAM and adrenaline in OHCA patients are both associated with an increase rate of ROSC, but only 31% of OHCA patients received prehospital AAM in Hefei, China. While in the United States, more than 80% of OHCA patients received prehospital AAM during the past decades [17]. A prospective observational study from the national OHCA registry reported that basic airway was used in 8.1% cases, supraglottic airway in 17.5%, and tracheal tube in 19.0%, whereas 5.1% (*n* = 1177) used more than one advanced airway in England during 2014 [18]. The rate of prehospital AAM in Hefei, China is lower than the rate in developed countries.

EMS systems have regional variability in prehospital AAM in the world. Could prehospital advanced airway improve survival in OHCA? There is controversy about the optimal approach to airway management in OHCA. Several nationwide retrospective observational studies in Japan have reported that prehospital AAM is associated with poor outcomes [5]. In a Korean nationwide retrospective cohort study, prehospital endotracheal intubation (ETI) was not associated with survival [6]. However, this study reported the prehospital AAM provision was associated with increased survival. The probability of ROSC in the advanced airway group was significantly higher than that in the no advanced airway group and increased by 66%.

The variations of those studies may be due to many factors. This includes type of AAM, process of AAM, proficiency and team CPR among EMS providers. Despite the different infrastructures of regional EMS systems in the world, time is of the essence in the provision of prehospital AAM. According to Izawa et al., based on their observational study, it was reported that resuscitation time bias in early ROSC was a limitation and early prehospital AAM provision was associated with neurological recovery in patients with OHCA [19]. A study reported that EMS providers with high exposure to ETI performance increased the survival rates of OHCA [20]. An association has been proved between ETI and significant interruptions in CPR [21]. Prehospital AAM without sufficient team CPR may contribute to increased rates of unnecessary interruption in chest compression performance. Each EMS provider has a predetermined role and emphasis is placed on high quality CPR in team CPR which is a choreographed approach. A meta-analysis study has reported that team CPR was associated with consistently increasing odds of survival to discharge and neurologic recovery of patients with OHCA [22].

Throughout the resuscitation effective bag-valve-mask airway may require more team resources compared with ventilation through a successfully placed advanced airway. Even so, sometimes the bag-valve-mask ventilation is not enough. This study suggested EMS gave the prehospital AAM in OHCA patients as soon as possible with minimal interruption of chest compression, and rescuers must understand the risks and benefits of AAM during resuscitation. The patient’s condition and rescuer’s airway management techniques affect these risks .

After attempting inserting an advanced airway device, the patient is provided with advanced life support, including the administration of adrenaline. The rationale for adrenaline administration is that it increases aortic diastolic pressure and improves coronary perfusion. The Adult Cardiac Arrest Algorithm has been updated to emphasize the early administration of adrenaline for patients with non-shockable rhythms [23]. The main purpose of drug therapy during cardiac arrest is to promote the recovery and maintenance of autonomic rhythm with perfusion.

There is uncertainty about the effect of adrenaline on neurological outcome, in addition to the variation in outcomes based on timing and initial rhythm. Study shows prehospital adrenaline administration by EMS is favorably associated with long-term neurological outcomes in patients with initial asystole and with long-term survival outcomes in those with pulseless electrical activity [24].

Currently, the study of prehospital AAM and adrenaline administration by EMS on outcomes of OHCA patients in China is not widely reported. This study found the OHCA witness rate and defibrillation implementation rate were low; meanwhile, emergency response time and prehospital time were longer than the golden treatment time of OHCA patients. (Table 1) However, this study found that the combination of advanced airway and adrenaline treatment significantly increased the probability of ROSC by 114% and survival to admission by 115%. Although delayed CPR may affect the prognosis of patients with tracheal intubation and adrenaline use, the advanced airway can provide stable ventilation, definite high oxygen supply and improve oxygenation. This may bring a better outcome of combination of advanced airway and adrenaline treatment. These findings suggest that EMS should try to use advanced airway and adrenaline as much as possible to improve the success rate of ROSC and the survival rate of OHCA patients.

Due to bystander CPR implementation rate, along with bystander defibrillation implementation rate being low, and regional EMS systems having different infrastructures in China, we suggest each agency should establish an individual strategy for prehospital managements and continuous quality assurance. and it will improve the survival of OHCA and provide the basis for further quality of EMS and research.

### Limitations

It is also the case that, there are limitations in the analysis. Firstly, no data on the prehospital AAM and adrenaline process were available, so the association between timing of the managements and outcomes after OHCA had not been discussed. Secondly, the start time of CPR was not accurately recorded. The duration of OHCA and emergency response time were longer than the golden treatment time of patients with cardiac arrest. Delayed CPR may affect the prognosis of patients with tracheal intubation and adrenaline use. Thirdly, different proficiency and team work efficiency of staff in multiple sub centers may affect the outcomes of resuscitation.

## Conclusion

The effects of prehospital advanced airway and the combined treatment of advanced airway and adrenaline in OHCA patients are both positively associated with outcome of ROSC. Moreover, the combined treatment of AAM and adrenaline can increase rate of survival to admission in OHCA patients. If combined treatment of advanced airway and adrenaline on the prehospital emergency rescue chain is improved and strengthened in EMS, the survival of OHCA patients may be greatly improved. It requires high quality prospective studies to provide a sound footing for measures to improve outcomes in this most critical of populations.

## Data Availability

The datasets used and analyzed during the current study are available from the corresponding author.

## Abbreviations

AAM: Advanced airway management
CPR: Cardiopulmonary resuscitation
OHCA: Out-of-hospital cardiac arrest
EMS: Emergency Medical Service
ROSC: Return of spontaneous circulation
DNAR: Do Not Attempt Resuscitation
PEA: Pulseless electrical activity
